# Pediatric Radial Neck Fracture: A Case Report Along With Surgical Tips on the Metaizeau Technique

**DOI:** 10.7759/cureus.56247

**Published:** 2024-03-15

**Authors:** Amit Kale, Ayush Taneja

**Affiliations:** 1 Orthopedics, Dr. D. Y. Patil Medical College, Hospital and Research Centre, Pune, IND

**Keywords:** paediatric radial neck fractures, intramedullary nailing, surgical tips, radial neck fracture, metaizeau

## Abstract

Radial neck fractures in children are an uncommon phenomenon. The Metaizeau technique for closed intramedullary nailing is a well-documented method for treating this type of fracture. We performed the Metaizeau technique for radial neck fracture fixation on a 10-year-old Indian male patient. The original method described by Metaizeau was followed, with surgical adjustments based on our experience to achieve a satisfactory result. This report provides the surgeon performing the Metaizeau technique with simple tips to assist in fracture reduction and fixation and avoid loss of reduction. These include oscillating movements of the T-handle for proximal progression of the nail/K-wire, gentle stabilizing counterforce over the radial head during entry into the proximal epiphysis, and moving the C-arm instead of the elbow during the nailing process for anteroposterior, oblique, and lateral imaging.

## Introduction

When viewed against all fractures in children, fractures of the radial neck only account for about 1% and about 5% to 10% of all traumatic injuries of the elbow seen in children [1]. Metaphyseal fractures and physeal fractures extending through the metaphysics (Salter-Harris II) are the most commonly seen anatomical types [2]. The most common mechanism of injury is a fall with the arm outstretched and forearm supinated along with an additional valgus thrust at the elbow, which causes radiocapitellar compression [3]. Judet et al. classify this fracture into four types based on displacement and angulation of the radial head [4]. The treatment of these fractures can be challenging, as a poor outcome can be associated with complications that include malunion, decreased range of motion, avascular necrosis, nonunion, cross-union, and even compartment syndrome. Radial neck fracture treatment is dependent on the age of the patient, degree of displacement, ability to improve the displacement, and the need for stabilization. In minimally displaced fractures, treatment is immobilization, for more displaced fractures, closed reduction and immobilization or open reduction are indicated. Owing to the higher potential of remodeling in children, a fracture with less than 30 degrees angulation is generally managed conservatively in the pediatric age group with closed reduction followed by immobilization in a plaster cast [5]. Displacement with angulations of over 30 degrees (Judet type III, IV) is an indication for surgical management [6]. In very young children (<10 years), angulations of upto 45 degrees can also be managed conservatively. Avascular necrosis of the radial head, restricted movements of the elbow joint, ectopic calcifications, and premature epiphyseal closure are some common complications seen with direct percutaneous K-wiring and open reduction techniques. Damage to the capsule and blood supply of the elbow joint are the main reasons for these complications. When feasible, the Metaizeau technique, which involves an intramedullary nailing procedure to achieve closed reduction is the preferred option, as it avoids damaging the capsule and radial head [7,8].

## Case presentation

**Figure 1:**
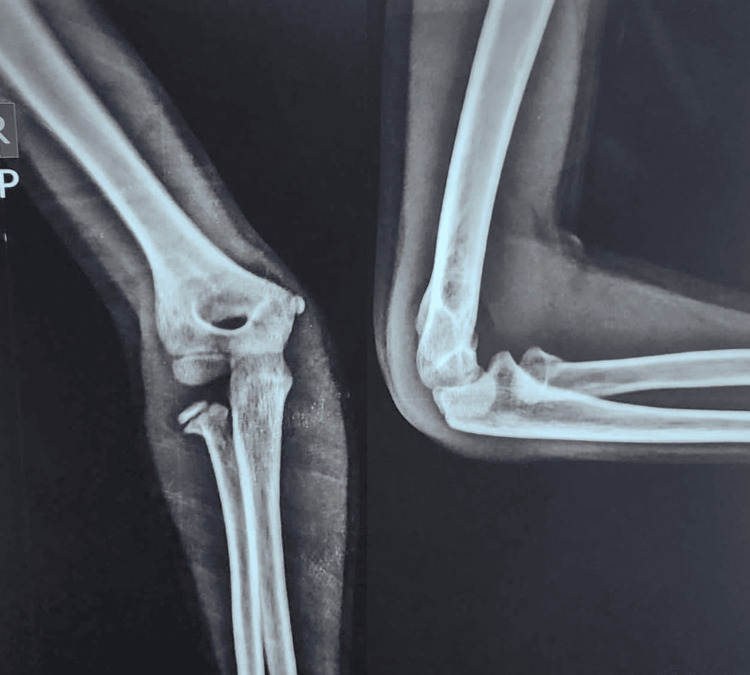
Preoperative anteroposterior and lateral radiographs showing a radial neck fracture (Judet type III)

A 10-year-old male presented to the emergency room, complaining of pain, swelling, and restricted movements of the right elbow following a history of a fall six hours ago. On examination, swelling and tenderness over the radial head were noted with no signs of neurovascular injury. The right elbow was then radiographed, which showed a Judet type III fracture of the radial neck (Figure 1).

**Figure 2:**
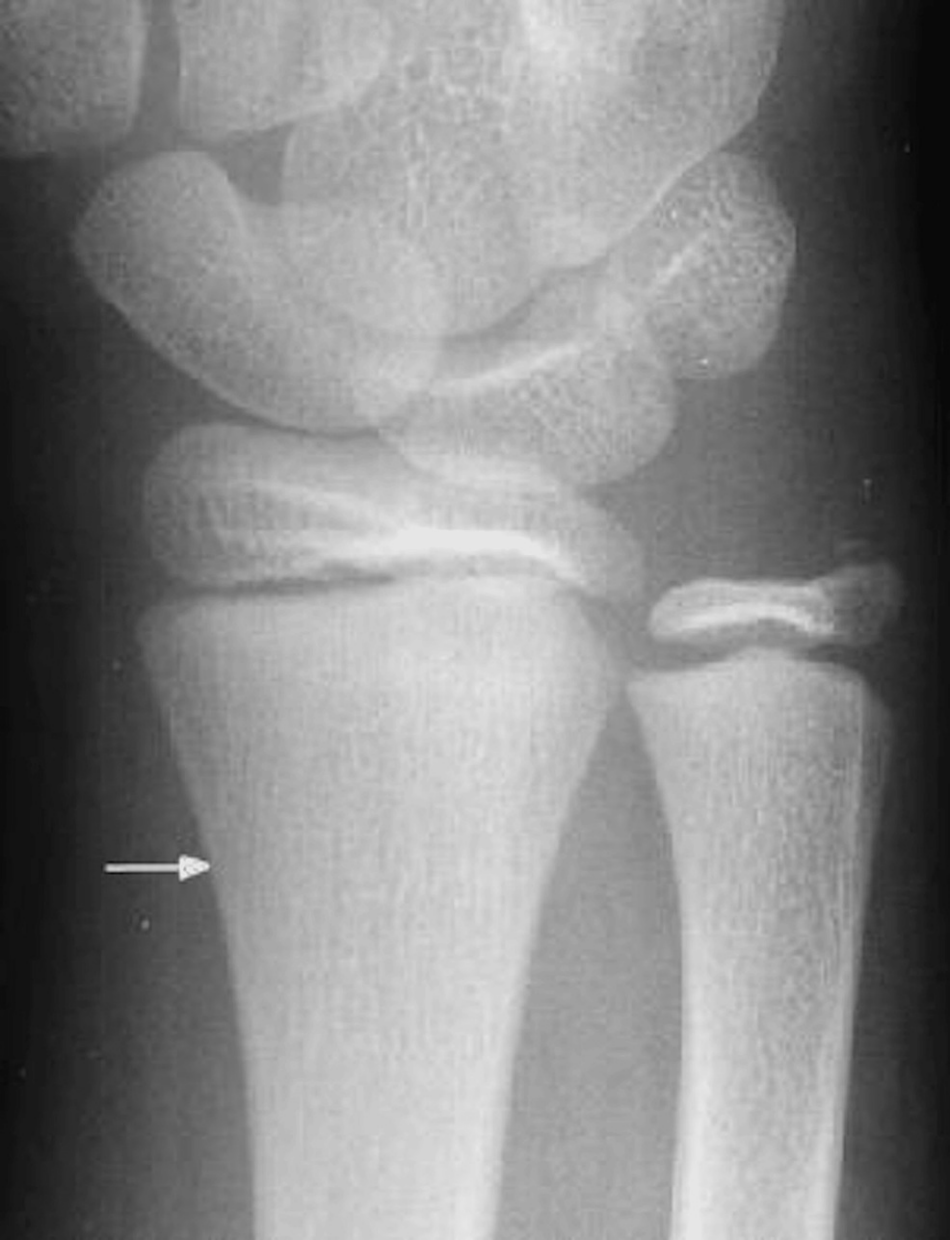
Entry point for K-wire insertion

An above-elbow posterior slab was applied to support the limb, and the patient was posted for surgery the next day. The patient was placed in the supine position on the operating table under general anesthesia. After the arm preparation, it was placed on an arm and hand surgery table. Patterson’s maneuver for closed reduction was initially attempted, which involved distal traction with the elbow in extension along with a varus stress on the supinated forearm and direct force on the radial head [9]. Due to inadequate reduction, we proceeded to perform the Metaizeau technique. The epiphyseal plate was marked, 2cm proximal to the plate, and a 1 cm incision was taken along the lateral aspect of the distal radial metaphysis (Figure 2).

**Figure 3:**
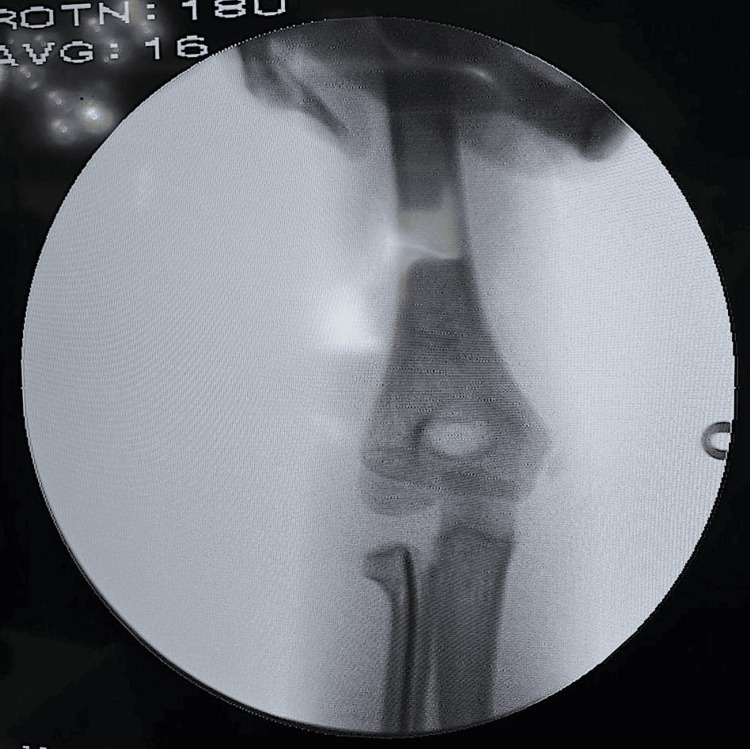
Intraoperative anteroposterior C-arm image following lifting of the proximal radial epiphysis

Care was taken to preserve the cutaneous branch of the radial nerve and superficial venous supply. Following the soft tissue dissection, the periosteum and brachioradialis insertion were stripped over an area adequate for nail insertion. The cortex was then penetrated using a bone awl with its tip directed along the medullary canal of the radius. A 1.8mm K-wire, with its distal 3 mm bent to an angle of 10 degrees, was then introduced and progressed proximally along the medullary canal. The original Metaizeau technique used a hammer for this step [7]; however, we find that using oscillating movements of the T-handle while stabilizing the forearm, allows the surgeon to navigate the medullary canal more effectively. The K-wire was then advanced to a point just inferior to the displaced epiphysis where the greatest tilt had been identified. Gentle taps of the mallet are then used to fix the point of the wire into the epiphysis and elevate it to be repositioned under the lateral condyle (Figure 3).

**Figure 4:**
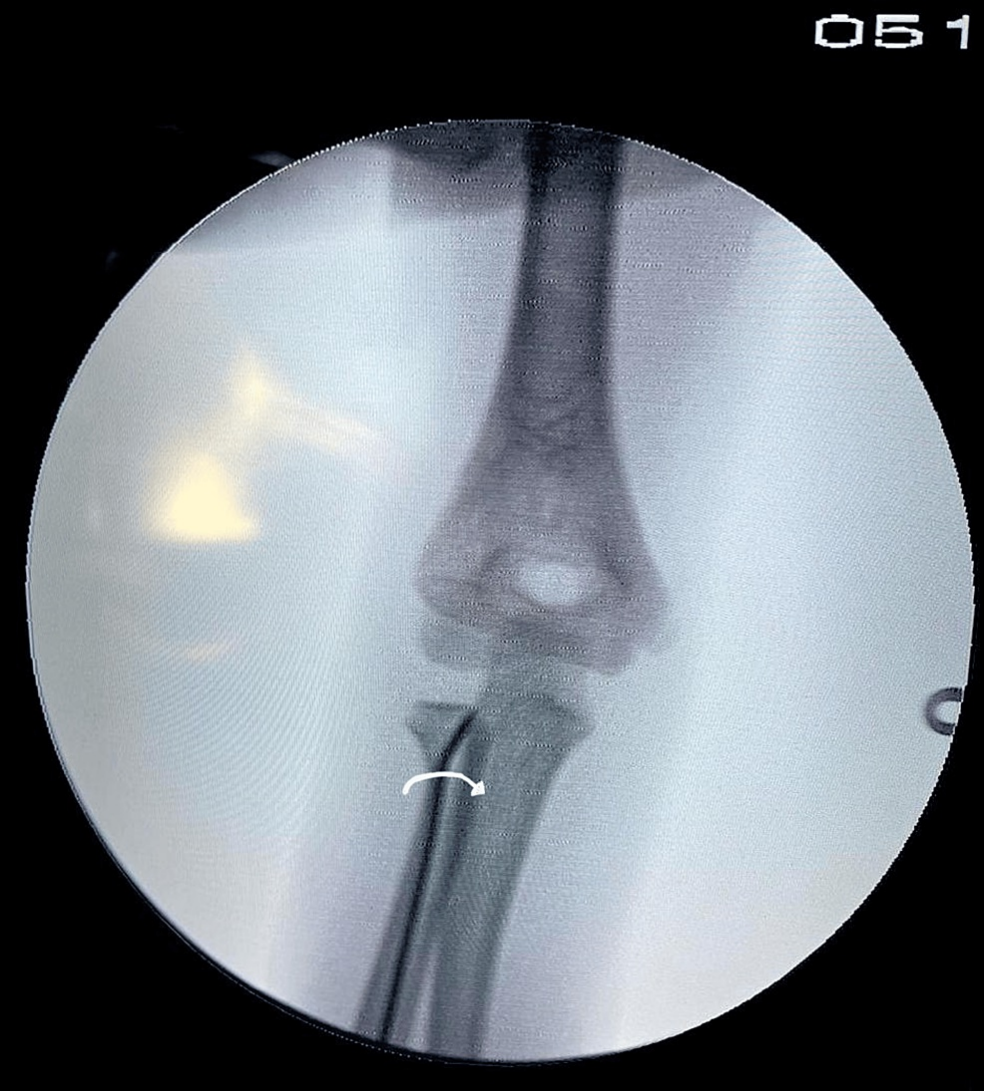
Intraoperative anteroposterior C-arm image following the turning of the K-wire 180 degrees along its long axis

We also advise applying a stabilizing counterforce proximally over the radial head to prevent further displacement while hammering. Following the correction of the tilt, lateral displacement was corrected by a 180-degree turn of the K-wire around its long axis (Figure 4).

**Figure 5:**
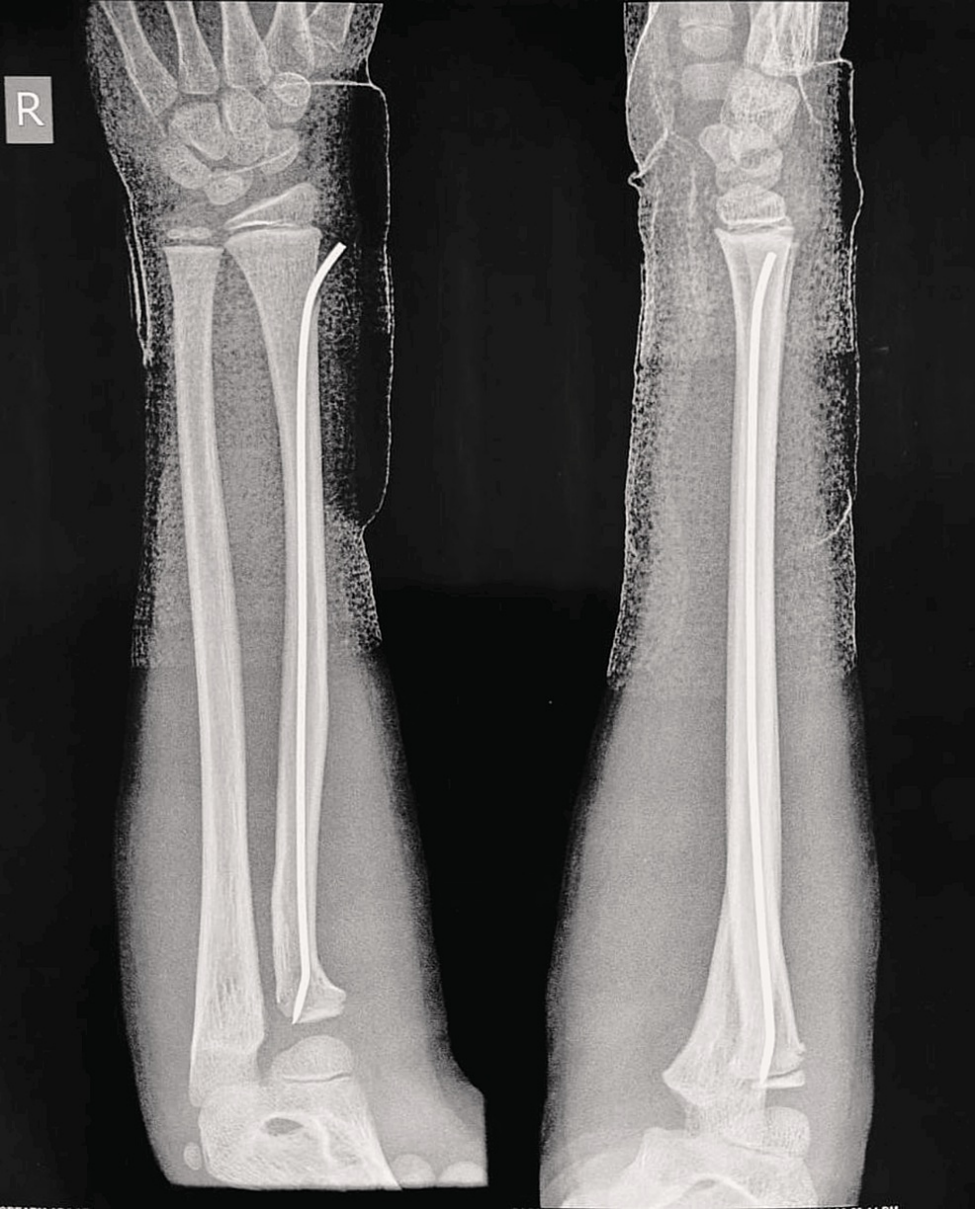
Postoperative anteroposterior and lateral radiographs following the Metaizeau procedure

Further gentle manipulation of the wire achieved a satisfactory reduction (Figure 5).

The reduction was then confirmed under the C-arm. Based on our experience, C-arm anteroposterior, lateral, and oblique images should be taken by rotating the C-arm rather than the patient's elbow, as this may cause a loss of reduction. The wire was then cut at its distal end and the incision site was then washed and closed. The patient was then kept in an above-elbow posterior slab for two weeks. Following this, mobilization was started gently and the patient achieved active elbow flexion of 120 degrees, supination of up to 60 degrees, and pronation of up to 45 degrees by 8 weeks, after which finally the K-wire removal was done. The patient then achieved a full elbow range of motion by 12 weeks and returned to his pre-trauma normal daily activities.

## Discussion

Radial neck fractures in the pediatric age group are a relatively rare phenomenon and their management can also be challenging if the displacement is large or associated complications, such as neurovascular compromise or compartment syndrome, are present. The age interval for pediatric radial neck fractures is 4 to 14 years, with the peak frequency being observed between 9 and 12 years of age [10]; this is due to the fragility of the conjugal epiphyseal cartilage before its complete ossification occurs at around 14-17 years of age [11]. This fracture in children is caused predominantly by an indirect mechanism that involves a fall on the outstretched hand with an extended elbow and additional valgus stress [3]. There is still controversy over the angle at which surgical intervention is advised. The general consensus among most authors is that angulations of more than 30° for children less than 10 years old and more than 15° for patients approaching skeletal maturity are indications for surgical management [12]. Younger patients are more likely to achieve satisfactory remodeling and hence the final treatment strategy is up to the surgeon. Percutaneous reduction and open surgery are among the other surgical options described in the literature [13]. Open surgical reduction and fixation methods are generally performed only on fractures with an angulation of more than 60° [14]. According to Metaizeau, large displacement/angulation features are also an indication for closed reduction [8]. This is supported by Tollet P [15]. An open surgical technique may help achieve anatomical reduction, however, epiphyseal vascularization may be compromised, which leads to an increased incidence of complications such as radial head necrosis [16]. In our case, the patient had presented with a type III Judet, radial neck fracture without any associated complications. We decided to take up closed intramedullary pinning as our preferred plan of action due to the fewer complications observed using this technique. The general method remained the same as described by Metaizeau [8]; however, we tweaked the procedure based on our experience to make it easier for the surgeon and to avoid loss of reduction.

## Conclusions

Radial neck fractures in the pediatric age group can be managed adequately with the Metaizeau technique. We found that oscillating movements of the T-handle along with minimal use of the mallet during proximal progression of the intramedullary nail can achieve a satisfactory result. While spearing the wire into the proximal radial epiphysis, a stable counterforce may be applied to the elbow by the assistant, which also ensures a stable entry into the epiphysis. Also, rotation of the C-arm for intraoperative radiographic imaging instead of movement of the elbow prevents loss of reduction during fracture fixation. These tips during the surgical procedure may help avoid loss of fracture reduction and help surgeons during the nailing process.
